# The combination of stem cell factor and granulocyte-colony stimulating factor for chronic stroke treatment in aged animals

**DOI:** 10.1186/2040-7378-4-25

**Published:** 2012-12-19

**Authors:** Chun-Shu Piao, Maria E Gonzalez-Toledo, Xi Gu, Li-Ru Zhao

**Affiliations:** 1 Department of Neurology, Louisiana State University Health Sciences Center, 1501 Kings Highway, Shreveport, LA, 71130, USA; 2 Department of Cellular Biology and Anatomy, Louisiana State University Health Sciences Center, 1501 Kings Highway, Shreveport, LA, 71130, USA; 3 Department of Pathology, Louisiana State University Health Sciences Center, 1501 Kings Highway, Shreveport, LA, 71130, USA

**Keywords:** Hematopoietic growth factor, SCF, G-CSF, Chronic stroke, Treatment, Elderly

## Abstract

**Background:**

Stroke occurs more frequently in the elderly population and presents the number one leading cause of persistent disability worldwide. Lack of effective treatment to enhance brain repair and improve functional restoration in chronic stroke, the recovery phase of stroke, is a challenging medical problem to be solved in stroke research. Our early study has revealed the therapeutic effects of stem cell factor (SCF) in combination with granulocyte-colony stimulating factor (G-CSF) (SCF+G-CSF) on chronic stroke in young animals. However, whether this treatment is effective and safe to the aged population remains to be determined.

**Methods:**

Cortical brain ischemia was produced in aged C57BL mice or aged spontaneously hypertensive rats. SCF+G-CSF or equal volume of vehicle solution was subcutaneously injected for 7 days beginning at 3–4 months after induction of cortical brain ischemia. Using the approaches of biochemistry assays, flow cytometry, pathology, and evaluation of functional outcome, several doses of SCF+G-CSF have been examined for their safety and efficiency on chronic stroke in aged animals.

**Results:**

All tested doses did not show acute or chronic toxicity in the aged animals. Additionally, SCF+G-CSF treatment in chronic stroke of aged animals mobilized bone marrow stem cells and improved functional outcome in a dose-dependent manner.

**Conclusions:**

SCF+G-CSF treatment is a safe and effective approach to chronic stroke in the aged condition. This study provides important information needed for developing a new therapeutic strategy to improve the health of older adults with chronic stroke.

## Background

Stroke is a cerebrovascular disease with the highest incidence occurring in those over the age of 60 [1]. Stroke also presents the number one cause of long-term disability in adults worldwide. A stroke is classified into 3 phases based on the pathological progression and timing after stroke onset: the acute phase, the subacute phase and chronic phase. The time frame of the three phases may be different for the individuals according to the location and the size of the infarction, the pathogenesis, cerebral vasculature response, and the age of the patients. Generally, acute stroke is the first 48 h after stroke onset, subacute stroke is 48 h to six weeks or to three months post-stroke, whereas chronic stroke is beyond three to six months after stroke onset.

Treatment for stroke is not well developed. Recombinant tissue plasminogen activator (rtPA) is the only US Food and Drug Administration-approved drug for treatment of ischemic stroke patients in the acute phase [2]. This thrombolytic therapy must be initiated within 4.5 h of stroke onset. Because of the limited time window for treatment and the potential for rtPA-induced intracerebral hemorrhage, [2,3] in fact, only 1-3% of stroke patients are able to receive this treatment [4]. As a result, more than 97% stroke patients lack a specific treatment; if they survive acute and subacute stroke, they must suffer from persistent disability and dependency because no effective treatment, other than physical therapy, is available for chronic stroke. Clearly, the effective intervention that can enhance stroke rehabilitation in the chronic phase is a critical need.

Stem cell factor (SCF) and granulocyte colony-stimulating factor (G-CSF) were originally discovered as hematopoietic growth factors two decades ago based on their effectiveness in supporting the survival and growth for hematopoietic stem cells or hematopoietic progenitor cells (HSCs/HPCs) [5,6]. SCF in combination with G-CSF (SCF+G-CSF) has been found to have synergistic effects in the mobilization of HSCs/HPCs from the bone marrow to the blood stream in both patients and laboratory animals [7]. In addition to the effects of SCF and G-CSF in bone marrow, accumulating evidence has also shown that SCF and G-CSF play a role in neuronal plasticity. SCF enhances neurite outgrowth in embryonic dorsal root ganglia [8,9]. cKit or SCF mutant mice show impaired long-term potentiation (LTP) and spatial learning [10,11]. G-CSF deficient mice also display cognitive impairment, LTP reduction, and poor neuronal networks in the hippocampus [12]. Recently, we have revealed that systemic administration of SCF+G-CSF but not SCF or G-CSF alone in chronic stroke leads to a stable and long-lasting functional improvement in 6–7 months old spontaneously hypertensive rats [13]. However, it remains unclear whether SCF+G-CSF treatment in chronic stroke is also suitable for the aged. To gain this knowledge is important because stroke has the highest incidence in the elderly. The aim of this study, therefore, is to determine the safety and efficiency of SCF+G-CSF in treatment of chronic stroke in aged animals.

## Methods

All the procedures in this study have been approved by the Institutional Animal Care and Use Committee and have been carried out in accordance with the National Institutes of Health Guide for the Care and Use of Laboratory Animals in the United States.

### Animals

Male C57BL mice at age of 16–19 months old and male spontaneously hypertensive rats (SHRs) at age of 11–13 months old were used in this study. These ages were chosen based on the different average lifespan of the two species: SHRs is approximately 18 months [14], while that of C57BL mice is 26 months [15]. Therefore, the 11–13 month-old SHRs and 16–18 month-old C57BL mice are equal to 61–72 years in humans, the population in which the highest incidence of stroke occurs. In addition, previous research has found aging-related changes in the neuronal biological function and neuronal metabolism in 12 month-old SHRs [16] and in 16–18 month-old C57BL mice [17,18].

### Animal model of cortical brain ischemia

The procedure for producing cortical brain ischemia in C67BL mice has been described elsewhere [19]. Briefly, aged C57BL mice were anesthetized with Avertin (2.4 mg/10g, i.p.) (Sigma-Aldrich, USA). The right common carotid artery (CCA) was ligated with a 6–0 silk suture through the midline incision in the neck, and the right middle cerebral artery (MCA) was coagulated with a coagulator (Bovie® AAron medical, USA) after a craniotomy was made between the right ear and eye. Cortical cerebral ischemia in male aged SHRs was similar to the procedures as stated in C67BL mice, and detailed information was provided in previous publications [20-22]. Briefly, after anesthetizing SHRs with Methohexital Sodium (50mg/kg, i.p.) (Eli Lilly, USA), the right CCA was permanently ligated with a 3–0 silk surgical suture and the right MCA was permanently ligated distal to the striatal branches with a 10–0 monofilament nylon suture. During the surgery, body temperature was monitored and maintained at 37°C by use of a heating pad coupled to a temperature regulator. Through the procedures stated above, the infarction was restricted in the right cortex in both C57BL mice and SHRs [19-22].

### Treatment

Recombinant mouse or recombinant rat SCF (PeproTech, USA) and recombinant human G-CSF (Amgen,USA) were subcutaneously injected for 5 days beginning at 3–4 months after induction of cortical brain ischemia. Six doses were tested in chronic stroke of aged mice: SCF (200 μg/kg/day) + G-CSF (50 μg/kg/day) (S+G 200/50), SCF (100 μg/kg/day) + G-CSF (25 μg/kg/day) (S+G 100/25), SCF (50 μg/kg/day) + G-CSF (25 μg/kg/day) (S+G 50/25), SCF (20μg/kg/day) + G-CSF (10 μg/kg/day) (S+G 20/10), SCF (10μg/kg/day) + G-CSF (5 μg/kg/day) (S+G 10/5), and SCF (5 μg/kg/day) + G-CSF (2.5 μg/kg/day) (S+G 5/2.5). Five mice were used for each group of tested doses. In addition, five doses were examined in aged SHRs of chronic stroke: SCF (100 μg/kg/day) + G-CSF (25 μg/kg/day) (S+G 100/25), SCF (50 μg/kg/day) + G-CSF (25 μg/kg/day) (S+G 50/25), SCF (20μg/kg/day) + G-CSF (10 μg/kg/day) (S+G 20/10), SCF (10μg/kg/day) + G-CSF (5 μg/kg/day) (S+G 10/5), and SCF (5 μg/kg/day) + G-CSF (2.5 μg/kg/day) (S+G 5/2.5). Three to eight SHRs were assigned to each group of tested doses.

### Flow cytometry

Four hours after the final injection of the 5-day treatment of SCF+G-CSF or saline, mice were anesthetized with Avertin (2.4 mg/10g, i.p.) (Sigma-Aldrich, USA), and blood was collected from the hearts. Blood cells were incubated with APC-conjugate anti-mouse CD117 antibody (anti-ckit, 1:100) or an equal amount of isotypematched APC antibodies (eBioscience, USA) on ice for 30 min. Red blood cells were then lysed with a FACS lysis buffer (BD Pharmingen, USA) and washed with phosphate buffered saline containing 0.5% fetal bovine serum. Thereafter, 5 x10^6^ cells were analyzed with a flow cytometer (FACSCalibur, BD, USA).

### Liver and kidney function assay

Blood samples were collected from the heart of mice or from the tail vein of SHRs 4h after the final injection of the 5-day treatment of SCF+G-CSF or saline and then sent to the Clinical Lab of our Medical Center for biochemical tests of liver and kidney functions. The liver enzymes including aspartate transaminase (AST), alanine transaminase (ALT), alkaline phosphatase (ALK phos), and gamma glutamyl transpeptidase (GGT) were used for testing liver function. Blood urea nitrogen (BUN) and serum creatinine were used for the kidney function test.

### Determination of liver and kidney weight and histological examination of liver and kidney injury

Four hours after the final injection of the 5-day treatment in mice or 14 weeks after the final treatment in SHRs, animals were anesthetized with Avertin (for mice, 2.4 mg/10g, i.p.) (Sigma-Aldrich, USA), or Methohexital Sodium (for SHRs, 50mg/kg, i.p.) (Eli Lilly ,USA), and the livers and kidneys were then removed. The weight of livers and kidneys was measured and corrected with the body weight. The livers and kidneys were then quickly cut into small pieces (2–3 mm thick) on ice. The liver and kidney samples were fixed in ice-cold 4% buffered-formaldehyde for 3 days and processed for paraffin embedding. Paraffin embedded sections (5μm thick) of liver and kidney were stained with hematoxylin and eosin (H&E). The H&E-stained sections were sent to the pathology, and drug-induced acute or chronic damage in the liver and kidney was examined in a blinded manner.

### Functional evaluation

Limb placement test [20,23,24] was used for evacuation of functional outcome in the SHRs and was performed in a blinded manner. In this test, forelimb and hindlimb placements were examined under eight different conditions. When the rat was gently pushed forward to the edge of a table or its limbs were placed near the edge of the table, the rat’s response was scored. For each test an animal received a score of 0 if it was unable to place its limb; a score of 1 if it was a partial and/or delayed (more than 2 sec) placement of its limb; or a score of 2 if it exhibited an immediate and correct placement of its limb. The maximum score is 16 for each side of the body. Score 0 means severe neurological deficits whereas score 16 presents no neurological deficits.

### Statistical analysis

One-way analysis of variance (ANOVA) was used for analyzing the data of liver and kidney functions. Kruskal-Wallis nonparametric analysis was used for determining the statistical difference of limb placement-test data. Multiple comparison-induced errors were adjusted with Bonferroni correction. P < 0.05 was considered statistically significant. Data were presented as mean±SE.

## Results

### The safety and efficiency of SCF+G-CSF treatment on chronic stroke in aged mice

Aged mice with experimental cortical stroke were randomly divided into 7 groups at 3–4 months after induction of cortical infarction: a saline control group and 6 groups with different tested doses.

**Figure 1:**
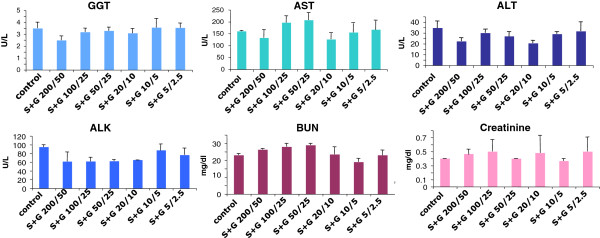
**Liver function tests (GGT, AST, ALT, and ALK) and kidney function tests (BUN and creatinine) after SCF+G-CSF treatment in chronic stroke with different doses in aged mice.** Blood samples were taken 4 hours after the final injection of SCF+G-CSF or saline. Noting that all biochemicals are not increased by SCF+G-CSF in any dose compared to saline controls. N=5. Mean ± SE.

**Figure 2:**
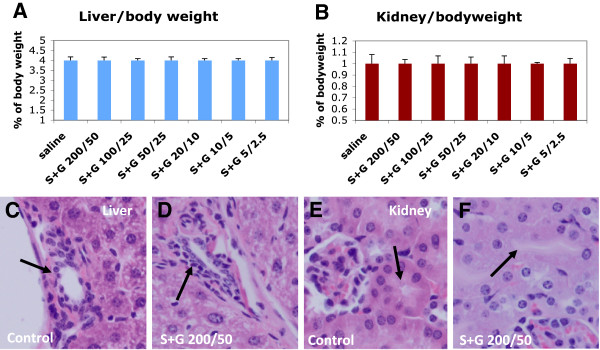
**Pathological examination of livers and kidneys after SCF+G-CSF treatment during chronic stroke in aged mice.** Livers and kidneys were removed 4 hours after the 5-day injection of SCF+G-CSF. (**A** &**B**) Liver and kidney weight that are corrected with body weight. Noting that there is no difference among the groups, indicating the treatment is not toxic. Mean ± SE, n=5. (**C**-**F**) Hematoxylin and eosin staining in liver and kidney sections. Noting that no damage is seen in both liver and kidney after a 5-day SCF+G-CSF treatment. S+G 200/50: SCF (200 μg/kg) in combination with G-CSF (50 μg/kg). Arrows in **C** &**D**: the bile canaliculus. Arrows in **E** &**F**: renal tubules.

To determine whether the treatment of SCF+G-CSF in aged chronic stroke mice has any toxic effects, the function and pathology of liver and kidney were examined because both liver and kidney are the most sensitive organs to drug toxicity. We observed that the levels of neither the liver function-related enzymes nor the kidney function-related chemicals were significantly increased by any dose of SCF+G-CSF (Figure 1). In addition, the weight of livers and kidneys in treated groups did not differ from those of the controls. Moreover, there was no drug-related damage in the bile canaliculi of the livers nor the renal tubules of the kidneys in any of the treated groups, including the high dose group (SCF 200μg/kg + G-CSF 50μg/kg) (Figure 2). These findings indicate that none of the doses tested here causes acute damage in either the livers or kidneys of aged mice. These data provide the evidence supporting that SCF+G-CSF at the doses ranging from S5+G2.5μg/kg to S200+G50μg/kg are the safe treatments in chronic stroke for aged mice.

**Figure 3:**
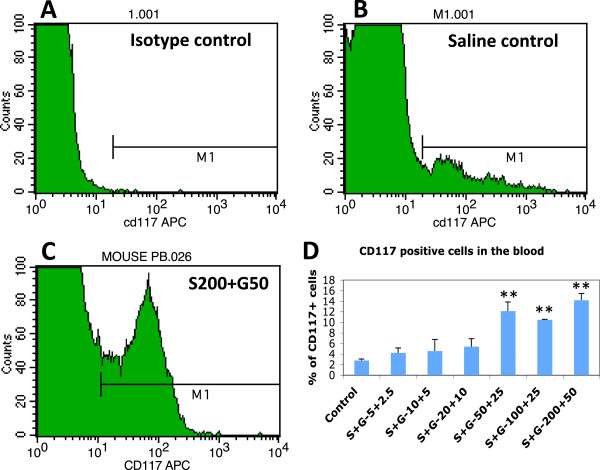
**Mobilization of bone marrow stem cells into the blood by SCF+G-CSF treatment during chronic stroke in aged C57BL mice.** Bone marrow stem cells in the blood were determined with an anti-CD117 antibody through flow cytometry. **A**-**C**: Flow cytometry data. **D**: Quantification data. Note that SCF+G-CSF treatment results in a significant elevation of circulating bone marrow stem cells in a dose-dependent manner. ** p < 0.01 as compared to saline controls. One-way ANOVA corrected with Bonferoni/Dunn. S+G: SCF+G-CSF. S200+G50: SCF (200 μg/kg) + G-CSF (50 μg/kg). All dose, μg/kg. Mean ± SE. N=5.

Our early study has revealed that SCF+G-CSF treatment in chronic stroke elevates the levels of bone marrow stem cells in the blood and that the bone marrow-derived progenitors are involved in SCF+G-CSF-induced enhancement of angiogenesis and neurogenesis in the peri-infarct cortex of the brains in young mice [19]. Therefore, we sought to determine the effective dose of SCF+G-CSF in treatment of chronic stroke in aged mice through testing the levels of bone marrow stem cells in the blood. Similar to the safety assays, blood samples were collected 4 h after the final injection of the 5-day treatment of SCF+G-CSF, and the levels of bone marrow stem cells were examined by flow cytometry with a CD117 antibody. We found that the levels of CD117^+^ bone marrow stem cells were increased in a dose dependent manner. In addition, a significant increase in the population of CD117^+^ bone marrow stem cells in the blood was seen only at the three higher doses (S200+G-50, S100+G25, and S50+G25) (Figure 3), indicating that these doses can effectively stimulate bone marrow stem cell mobilization in aged mice during chronic stroke.

### The safety and efficiency of SCF+G-CSF treatment on chronic stroke in aged spontaneously hypertensive rats

Most stroke patients have a long history of hypertension. As mentioned earlier, stroke has the highest incidence in the aged population. To consider the clinical relevance, we utilized the aged spontaneously hypertensive rats (SHRs) to make cortical infarcts (stroke). Using such a model, the safety and effectiveness of selected 5 doses of SCF+G-CSF in treatment of chronic stroke were examined.

**Figure 4:**
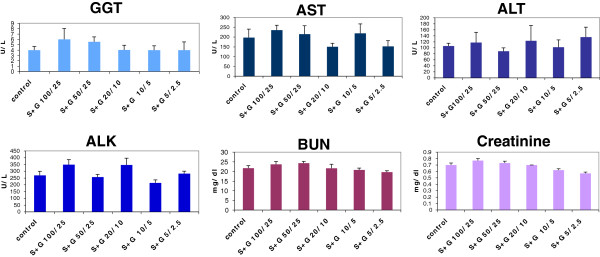
**Liver function tests (GGT, AST, ALT, and ALK) and kidney function tests (BUN and Creatinine) after SCF+G-CSF treatment with different doses during chronic stroke in aged SHRs.** Blood samples were taken from the tail vein 4 hours after the final injection of SCF+G-CSF or saline. Noting that no difference is seen between controls and any of the 5 dose treatments for all tests (p>0.05). N=3-8, mean±SE.

**Figure 5:**
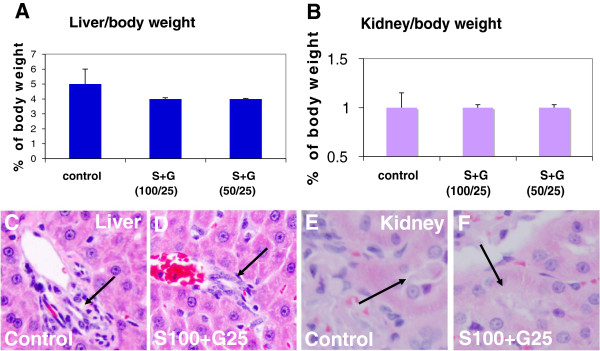
**Pathological examination of liver and kidney after SCF+G-CSF treatment during chronic stroke in aged SHRs.** Livers and kidneys were taken 14 weeks after the SCF+G-CSF treatment. (**A** &**B**) Liver and kidney weight. Noting that there are no differences among the groups, indicating that the treatment has no toxic effects on the liver and kidney. (**C**-**F**) hematoxylin and eosin staining in liver and kidney sections. Noting that no damage is seen in both liver and kidney after SCF+G-CSF treatment. Arrows in **C** &**D**: the bile canaliculus. Arrows in **E** &**F**: renal tubules. N= 3–8. Mean ± SE.

First of all, we determined the toxicity of each dose through functional and pathological examinations for the liver and kidney. The experimental design was the same as stated earlier in the study of aged mice. We observed the same results as in the aged mice that none of the tested doses caused an increase in liver function-related enzymes or in kidney function-related chemicals (Figure 4), indicating that the SCF+G-CSF treatment in chronic stroke does not cause acute damage in the liver and kidney in aged SHRs. In addition, we also tested drug-related chronic damage 14 weeks after SCF+G-CSF treatment. We found that the liver and kidney weights of any of the treated rats were no different from those of the controls (Figure 5 A & B illustrating representative data for the two higher doses). Further, pathological examination of the liver and kidney sections showed that the bile canaliculi in the livers and renal tubules in the kidneys, the two areas usually demonstrating drug damage, did not show any damage in any treated animals (Figure 5 C-F), indicating no chronic damage in the livers and kidneys after SCF+G-CSF treatment in the aged SHRs of chronic stroke. These data suggest that all the tested doses of SCF+G-CSF are safe for treating aged SHRs in the chronic phase.

**Figure 6:**
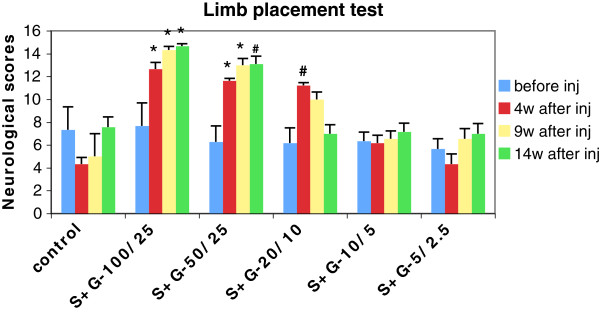
**Functional recovery evaluation after SCF+G-CSF treatment during chronic stroke in aged SHRs.** Noting that long-lasting functional improvement is seen in SCF+G-CSF (S+G) at doses of 100μg/kg SCF with 25μg/kg G-CSF (S+G-100/25) and 50μg/kg SCF with 25μg/kg G-CSF (S+G-50/25). Treatment with 20μg/kg SCF with 10μg/kg G-CSF (S+G-20/10) only shows temporal improvement in functional outcome. # p < 0.05 and * p < 0.01 vs. control and the two lower doses: S+G-10/5μg/kg and S+G-5/2.5 μg/kg. N=3-8. Mean ± SE.

In our early study, we demonstrated that SCF+G-CSF treatment in chronic stroke induced a stable and long-lasting functional improvement in SHRs at relatively young age [13]. Can this treatment also have therapeutic effects in aged SHRs? If so, which is the effective dose? To address these questions, we tested five doses of SCF+G-CSF in chronic stroke of aged SHRs. The stroke SHRs were randomized to receive the five doses of SCF+G-CSF or equal volumes of saline beginning at 3–4 months post-stroke. Functional recovery was evaluated with limb placement test 4, 9, and 14 weeks after the final treatment. We found that a stable and long-lasting functional improvement was seen in the rats treated with the two higher doses (S100+G25 and S50+G25). In addition, S20+G10 only caused a temporary functional recovery, whereas the two doses lower than S20+G10 did not show functional improvement (Figure 6). This observation suggests that SCF+G-CSF-induced functional recovery is dose-dependent.

## Discussion

In this study we have examined several doses of SCF+G-CSF in chronic stroke in the aged mice and SHRs and demonstrated that SCF+G-CSF treatment in chronic stroke of the aged has no toxic effects. In addition, we have also noted a dose-dependent therapeutic effect of SCF+G-CSF on chronic stroke in the aged animal models.

Stroke highly attacks aged population. About two-thirds of strokes occur in elderly people over age 65 in the United States [1]. It has been concerned that advancing age may alter biological and physiological functions, and these changes may cause a different response to drug treatment [25,26]. In this study we used aged mice and SHRs at which the age is equal to 61–72 years in humans to determine the safety and effectiveness of SCF+G-CSF in chronic stroke. Using liver and kidney function assays and pathological examination of liver and kidney, drug-related acute damage (4 h after 5-day treatment) or drug-related chronic injury (14 weeks after treatment) in both liver and kidney was not found in those of SCF+G-CSF-treated animals, suggesting that this treatment in chronic stroke in the aged population is a safe approach.

Convincing evidence has supported that SCF in combination with G-CSF can synergistically mobilize HSCs/HPCs from bone marrow to the blood, resulting in a robust elevation of HSCs/HPCs in the blood stream in humans, rodents, dogs and nonhuman primates [7]. One of our earlier studies suggested that SCF+G-CSF-induced mobilization of HSCs/HPCs contributed to brain repair in the chronic stroke as the great number of bone marrow-derived cells were found in SCF+G-CSF-treated animals and SCF+G-CSF augments bone marrow-derived cerebral endothelial cells and bone marrow-derived neurons [19]. Use of mobilization of HSCs/HPCs as an index, we found that SCF+G-CSF at the dose ranging from 50μg/kg SCF+25μg/kg G-CSF to 200μg/kg SCF+50μg/kg G-CSF was sufficient to mobilize HSCs/HPCs during chronic stroke in aged mice.

Stroke model produced in aged SHRs provides a clinical-relevant animal model of stroke because hypertension is the most important risk factor for stroke in humans [27]. Chronic hypertension causes extensive changes in the cerebrovascular bed [28,29]. Occlusion of the middle cerebral artery distal to the striatal branch and of the ipsilateral common carotid artery in SHRs, the rat model of stroke used for this study, leads to a more consistent and larger infarction in the cortex than in normotensive rats because of inadequate blood flow through collateral vessels in the SHRs [13,20,22,29-35]. In addition to the consistent infarction, this model also induces permanent deficits in somatosensorimotor function that last up to the chronic phase of stroke. Furthermore, this model has no problem for long-term survival [13,20,22,23,33]. Using this model, we have demonstrated that treatment of chronic stroke with SCF+G-CSF at the dose of 100μg/kg SCF + 25μg/kg G-CSF and 50μg/kg SCF + 25μg/kg G-CSF causes a relatively stable and long-lasting functional improvement in aged SHRs. Although it remains poorly understood how SCF+G-CSF repairs an aged brain in chronic stroke, our recent findings suggest that SCF+G-CSF-induced reestablishment of stable neuronal networks in the peri-infarct cortex may play an important role in the SCF+G-CSF-induced functional recovery (Cui et al., unpublished observation). The limitation of the functional evaluation in this study is that we did not perform the limb placement test before induction of cortical brain ischemia to gain the baseline performance for each SHR.

It is worth noting that SCF+G-CSF would be easily to be translated into clinical trials, as this therapy has been proven safe and effective to mobilize HSCs/HPCs for bone marrow transplantation in cancer patients after chemotherapy [36-38].

In summary, the present study has demonstrated that the combination of two hematopoietic growth factors, SCF and G-CSF, is a safe and effective treatment for chronic stroke in the aged condition. This observation provides new knowledge to assist in developing a new therapeutic strategy for chronic stroke.

## Abbreviations

ALK: Alkaline phosphatase; phos ALT: Alanine transaminase; AST: Aspartate transaminase; BUN: Blood urea nitrogen; GGT: Gamma glutamyl transpeptidase; G-CSF: Granulocyte-colony stimulating factor; SCF: Stem cell factor; SHR: Spontaneously hypertensive rat.

## Competing interests

The authors declare no competing interests.

## Authors’ contributions

CSP and MEG-T performed experiment. XG examined the pathology of livers and kidneys, CSP and LRZ analyzed data, and LRZ designed the experiment and prepared the manuscript. All authors read and approved the final manuscript.

## References

[B1] Roger VL Go AS Lloyd-Jones DM Benjamin EJ Berry JD Borden WB Heart Disease and Stroke Statistics–2012 Update: A Report From the American Heart Association Circulation 2012 125 e2 e200 22179539 10.1161/CIR.0b013e31823ac046 PMC4440543

[B2] Hill MD Hachinski V Stroke treatment: time is brain Lancet 1998 352 SIII10 4 9803956 10.1016/s0140-6736(98)90088-5

[B3] Pfefferkorn T Rosenberg GA Closure of the blood–brain barrier by matrix metalloproteinase inhibition reduces rtPA-mediated mortality in cerebral ischemia with delayed reperfusion Stroke 2003 34 2025 2030 10.1161/01.STR.0000083051.93319.28 12855824

[B4] Warlow C Sudlow C Dennis M Wardlaw J Sandercock P Stroke Lancet 2003 362 1211 1224 10.1016/S0140-6736(03)14544-8 14568745

[B5] Welte K Platzer E Lu L Gabrilove JL Levi E Mertelsmann R Moore MA Purification and biochemical characterization of human pluripotent hematopoietic colony-stimulating factor Proc Natl Acad Sci USA 1985 82 1526 1530 10.1073/pnas.82.5.1526 3871951 PMC397296

[B6] Zsebo KM Wypych J McNiece IK Lu HS Smith KA Karkare SB Identification, purification, and biological characterization of hematopoietic stem cell factor from buffalo rat liver–conditioned medium Cell 1990 63 195 201 10.1016/0092-8674(90)90300-4 2208278

[B7] Duarte RF Frank DA The synergy between stem cell factor (SCF) and granulocyte colony-stimulating factor (G-CSF): molecular basis and clinical relevance Leuk Lymphoma 2002 43 1179 1187 10.1080/10428190290026231 12152985

[B8] Hirata T Morii E Morimoto M Kasugai T Tsujimura T Hirota S Kanakura Y Nomura S Kitamura Y Stem cell factor induces outgrowth of c-kit-positive neurites and supports the survival of c-kit- positive neurons in dorsal root ganglia of mouse embryos Development 1993 119 49 56 7506140 10.1242/dev.119.1.49

[B9] Hirata T Kasugai T Morii E Hirota S Nomura S Fujisawa H Kitamura Y Characterization of c-kit- positive neurons in the dorsal root ganglion of mouse Brain Res Dev Brain Res 1995 85 201 211 10.1016/0165-3806(94)00205-e 7541320

[B10] Katafuchi T Li AJ Hirota S Kitamura Y Hori T Impairment of spatial learning and hippocampal synaptic potentiation in c-kit mutant rats Learn Mem 2000 7 383 392 10.1101/lm.33900 11112797 PMC311355

[B11] Motro B Wojtowicz JM Bernstein A van der Kooy D Steel mutant mice are deficient in hippocampal learning but not long-term potentiation Proc Natl Acad Sci USA 1996 93 1808 1813 10.1073/pnas.93.5.1808 8700840 PMC39863

[B12] Diederich K Sevimli S Dörr H Kösters E Hoppen M Lewejohann L The role of granulocyte-colony stimulating factor (G-CSF) in the healthy brain: a characterization of G-CSF-deficient mice J Neurosci 2009 2009 29 11572 11581 19759304 10.1523/JNEUROSCI.0453-09.2009 PMC6665757

[B13] Zhao LR Berra HH Duan WM Singhal S Mehta J Apkarian AV Kessler JA Beneficial effects of hematopoietic growth factor therapy in chronic ischemic stroke in rats Stroke 2007 38 2804 2811 10.1161/STROKEAHA.107.486217 17761920

[B14] Udenfriend S Bumpus FM Foster HL Rascher W, Clough D, Ganten D Spontaneously hypertensive rats: guidelines for breeding, care, and use Hypertensive mechanisms 1982 Stuttgart: Schattauer Verlag 781 801

[B15] National Research Council Mammalian Models for Research on Aging 1981 Washington DC: National academy press 48

[B16] Knox CA Yates RD Chen I Brain aging in normotensive and hypertensive strains of rats II. Ultrastructural changes in neurons and glia. Acta Neuropathol 1980 52 7 15 10.1007/BF00687223 6254320

[B17] Vogel RW Ewers M Ross C Gould TJ Woodruff-Pak DS Age-related impairment in the 250- millisecond delay eyeblink classical conditioning procedure in C57BL/6 mice Learn Mem 2002 9 321 336 10.1101/lm.50902 12359840 PMC187122

[B18] Williams WM Stadtman ER Moskovitz J Ageing and exposure to oxidative stress in vivo differentially affect cellular levels of PrP in mouse cerebral microvessels and brain parenchyma Neuropathol Appl Neurobiol 2004 30 161 168 10.1111/j.1365-2990.2003.00523.x 15043713

[B19] Piao CS Gonzalez-Toledo ME Xue YQ Duan WM Terao S Granger DN Kelley RE Zhao LR The role of stem cell factor and granulocyte-colony stimulating factor in brain repair during chronic stroke J Cereb Blood Flow Metab 2009 29 759 770 10.1038/jcbfm.2008.168 19209180

[B20] Zhao LR Duan WM Reyes M Keene CD Verfaillie CM Low WC Human bone marrow stem cells exhibit neural phenotypes and ameliorate neurological deficits after grafting into the ischemic brain of rats Exp Neurol 2002 174 11 20 10.1006/exnr.2001.7853 11869029

[B21] Zhao LR Navalitloha Y Singhal S Mehta J Piao CS Guo WP Kessler JA Groothuis DR Hematopoietic growth factors pass through the blood–brain barrier in intact rats Exp Neurol 2007 204 569 573 10.1016/j.expneurol.2006.12.001 17307165 PMC3099460

[B22] Zhao LR Singhal S Duan WM Mehta J Kessler JA Brain repair by hematopoietic growth factors in a rat model of stroke Stroke 2007 38 2584 2591 10.1161/STROKEAHA.106.476457 17656664

[B23] Ohlsson AL Johansson BB Environment influences functional outcome of cerebral infarction in rats Stroke 1995 26 644 649 10.1161/01.STR.26.4.644 7709412

[B24] De Ryck M Van Reempts J Borgers M Wauquier A Janssen PA Photochemical stroke model: flunarizine prevents sensorimotor deficits after neocortical infarcts in rats Stroke 1989 20 1383 1390 10.1161/01.STR.20.10.1383 2799870

[B25] Gurwitz JH Avorn J The ambiguous relation between aging and adverse drug reactions Ann Intern Med 1991 114 956 966 2024864 10.7326/0003-4819-114-11-956

[B26] Turnheim K Drug dosage in the elderly. Is it rational? Drugs Aging 1998 13 357 379 10.2165/00002512-199813050-00003 9829164

[B27] Johansson BB Auer LM Sayama I Reaction of pial arteries and veins to hypercapnia in hypertensive and normotensive rats Stroke 1985 16 320 323 10.1161/01.STR.16.2.320 3975971

[B28] Johansson BB Cerebral vascular bed in hypertension and consequences for the brain Hypertension 1984 6 III81 III86 10.1161/01.HYP.6.6_Pt_2.III81 6519759

[B29] Barone FC Price WJ White RF Willette RN Feuerstein GZ Genetic hypertension and increased susceptibility to cerebral ischemia Neurosci Biobehav Rev 1992 16 219 233 10.1016/S0149-7634(05)80182-4 1630732

[B30] Coyle P Different susceptibilities to cerebral infarction in spontaneously hypertensive (SHR) and normotensive Spraque-Dawley rats Stroke 1986 17 520 525 10.1161/01.STR.17.3.520 3715954

[B31] Duverger D MacKenzie ET The quantification of cerebral infarction following focal ischemia in the rat: influence of strain, arterial pressure, blood glucose concentration, and age J Cereb Blood Flow Metab 1988 8 449 461 10.1038/jcbfm.1988.86 2968987

[B32] Grabowski M Nordborg C Brundin P Johansson BB Middle cerebral artery occlusion in the hypertensive and normotensive rat: a study of histopathology and behaviour J Hypertens 1988 6 405 411 3385206

[B33] Stroemer RP Kent TA Hulsebosch CE Neocortical neural sprouting, synaptogenesis, and behavioral recovery after neocortical infarction in rats Stroke 1995 26 2135 2144 10.1161/01.STR.26.11.2135 7482662

[B34] Zhao LR Mattsson B Johansson BB Environmental influence on brain-derived neurotrophic factor messenger RNA expression after middle cerebral artery occlusion in spontaneously hypertensive rats Neuroscience 2000 97 177 184 10.1016/S0306-4522(00)00023-3 10771349

[B35] Zhao LR Risedal A Wojcik A Hejzlar J Johansson BB Kokaia Z Enriched environment influences brain-derived neurotrophic factor levels in rat forebrain after focal stroke Neurosci Lett 2001 305 169 172 10.1016/S0304-3940(01)01837-7 11403932

[B36] Facon T Harousseau JL Maloisel F Attal M Odriozola J Alegre A Schroyens W Hulin C Schots R Marin P Guilhot F Granena A De Waele M Pigneux A Méresse V Clark P Reiffers J Stem cell factor in combination with filgrastim after chemotherapy improves peripheral blood progenitor cell yield and reduces apheresis requirements in multiple myeloma patients: a randomized, controlled trial Blood 1999 94 1218 1225 10438709

[B37] Stiff P Gingrich R Luger S Wyres MR Brown RA LeMaistre CF Perry J Schenkein DP List A Mason JR Bensinger W Wheeler C Freter C Parker WRL Emmanouilides C A randomized phase 2 study of PBPC mobilization by stem cell factor and filgrastim in heavily pretreated patients with Hodgkin’s disease or non-Hodgkin’s lymphoma Bone Marrow Transplant 2000 26 471 481 10.1038/sj.bmt.1702531 11019835

[B38] To LB Bashford J Durrant S MacMillan J Schwarer AP Prince HM Gibson J Lewis I Swart B Marty J Rawling T Ashman L Charles S Cohen B Successful mobilization of peripheral blood stem cells after addition of ancestim (stem cell factor) in patients who had failed a prior mobilization with filgrastim (granulocyte colony-stimulating factor) alone or with chemotherapy plus filgrastim Bone Marrow Transplant 2003 31 371 378 10.1038/sj.bmt.1703860 12634728

